# Names of the Graduating Class of Rush Medical College

**Published:** 1860-05

**Authors:** 


### NAMES OF THE GRADUATING CLASS

OF

RUSH MEDICAL COLLEGE—SESSION, 1859—60.

| | |
|---|---|
| D. Orson B. Adams, Ill. | Leigh R. Holmead, Ill. |
| John J. M. Angier, Wis. | M. A. Isaac, " |
| John T. Bellington, " | Hiram C. Luce, " |
| Frederick Bartells, Ill. | Percy McAlpin, " |
| John B. Baker, " | Philip Matthei, " |
| Ed. L. H. Barr, " | Wm. F. Osborn, " |
| Hiram Carnahan, Iowa. | Geo. W. Richards, " |
| John M. C. Damron, Ill. | Edward Thomas, " |
| Henry Durham, Wis. | Vincent S. Thompson," |
| B. Irvin Dunn, Ill. | J. S. Underwood, " |
| R. M. Elliott, " | William V. Wiles, " |
| John B. Felker, " | Calvin Wheeler, " |
| A. M. Golliday, " | William Irwin, Wis. |
| Jethro A. Hatch, " | Sam'l A. Sheldon, " |
| Danl. Kirkpatrick, Ind. | C. M. Smith, " |
| Thos. J. Fritts, " | John E. Ennis, Iowa. |
| John Dancer, " | Robert B. Ray, Mo. |
| James Thompson, " | James F. Spain, Ohio. |

