# Correction

**Published:** 1860-09

**Authors:** 


Correction.—In the list of graduates of Rush Medical College, for the session of 1859–60, published in the May number of the Journal, a typographical error occurred in the name of Dr. Barry; it should read, Ed. L. H. Barry.

